# Injecting on the Island: a qualitative exploration of the service needs of persons who inject drugs in Prince Edward Island, Canada

**DOI:** 10.1186/1477-7517-11-10

**Published:** 2014-03-04

**Authors:** Jessica M McCutcheon, Melanie A Morrison

**Affiliations:** 1 Department of Psychology, University of Saskatchewan, 9 Campus Drive, Arts Building, Saskatoon, Saskatchewan S7N 5A5, Canada

**Keywords:** Injection drug use, Harm reduction, Service provision, Syringe access, Canada

## Abstract

**Background:**

Few studies have investigated the service needs of persons who inject drugs (PWID) who live in less populated regions of Canada. With access to fewer treatment and harm reduction services than those in more urban environments, the needs of PWID in smaller centres may be distinct. As such, the present study examined the needs of PWID in Prince Edward Island (PEI), the smallest of Canada's provinces.

**Methods:**

Eight PWID were interviewed about the services they have accessed, barriers they faced when attempting to access these services, and what services they need that they are not currently receiving.

**Results:**

Participants encountered considerable barriers when accessing harm reduction and treatment services due to the limited hours of services, lengthy wait times for treatment, and shortage of health care practitioners. They also reported experiencing considerable negativity from health care practitioners. Participants cited incidences of stigmatisation, and they perceived that health care practitioners received insufficient training related to drug use. Recommendations for the improvement of services are outlined.

**Conclusions:**

The findings indicate that initiatives should be developed to improve PWID's access to harm reduction and treatment services in PEI. Additionally, health care practitioners should be offered sensitisation training and improved education on providing services to PWID. The findings highlight the importance of considering innovative alternatives for service provision in regions with limited resources.

## Background

To date, the majority of the research examining the needs of persons who inject drugs (PWID) in Canada has been conducted in large urban centres, specifically, Toronto, Vancouver, and Montreal. The limited empirical attention paid to Canadian PWID who live in rural or remote areas is disconcerting for three reasons: (1) studies investigating prevalence rates of injection drug use in rural areas outside of Canada suggest that there may be an increasing need for services in smaller regions or remote areas [1-4], (2) less populated regions may encounter unique institutional challenges when providing harm reduction and other treatment services due to the costs associated with meeting the needs of a geographically dispersed population and with recruiting and retaining skilled health care practitioners [5,6], and (3) at an individual level, PWID may experience increased stigma and concern about confidentiality and anonymity due to the area in which they reside, as well as significant financial burdens if attempting to procure specialised health care services [7-9].

The purpose of the present study, therefore, was to explore the service needs of self-identifying PWID in the smallest of Canada's provinces, Prince Edward Island (PEI). Set on the east coast of Canada, an area that contains many rural and remote centres due to its geographical make-up, PEI is referred to as one of the Atlantic provinces, as is Newfoundland, New Brunswick, and Nova Scotia. At present, the authors are aware of only three published studies [9-11] that have investigated injection drug use in Atlantic Canada, the first of which is centred in Newfoundland [10]. Set in St. John's, the capital city of Newfoundland, the participants in the study of Gustafson et al. [10] were 44 PWID (29 of whom were surveyed, 15 interviewed), along with 34 service providers, nurses, government workers, and policy makers. Results indicated the presence of significant barriers for PWID when trying to access health services in a smaller Canadian centre. The researchers speculated that small urban centres such as St. John's, Newfoundland do not have the economic advantages of large cities and, thus, are unable to adequately fund programmes and services for PWID. As the first published study examining the needs of PWID in Atlantic Canada, the study of Gustafson et al. [10] provides invaluable information on potential barriers encountered by PWID in small urban centres. However, it is important to point out that the experiences of PWID in St. John's, Newfoundland may not be representative of PWID in other small Canadian centres given the geographic isolation and economic distinctiveness of the location.^a^

The two most recently published studies [9,11], focussing on PWID residing in the Atlantic region, interviewed PWID from all four of Canada's easternmost provinces (Newfoundland, New Brunswick, Nova Scotia, and PEI). Parker et al. [9] and Jackson et al. [11], two articles derived from the same large-scale study, interviewed 115 PWID to bring insight to how social and familial relationships affect participants' drug-related practices. By interviewing PWID residing in non-urban areas throughout Atlantic Canada, in addition to urban locations, Parker et al. [9] were able to highlight the unique barriers encountered by PWID whose geographic distance from harm reduction services precluded the same degree of service access that could be expected by PWID living in urban centres. The researchers found that PWID faced many obstacles when accessing harm reduction services, primarily in the form of perceived stigma from health care practitioners and pharmacists and, for rural PWID specifically, the unavailability of services within their region. In fact, PWID living outside urban centres who did not receive outreach services were found to encounter greater challenges in accessing and disposing sterile and used injecting equipment, respectively, and were unable to benefit from the additional resources offered by harm reduction programmes (e.g. referrals, social support, and food). Drawing upon the same sample of 115 PWID in Atlantic Canada, Jackson et al. [11] specifically discuss the role of family members in providing harm reduction support to PWID. By exploring the role of familial relationships from the perspective of the PWID, as opposed to the family members, the researchers present an in-depth analysis of the opportunities and barriers resulting from this innovative approach to harm reduction. The researchers note that harm reduction interventions initiated by family members may be particularly important for PWID in rural areas who may have no, or limited, access to services.

While Jackson et al. [11] and Parker et al. [9] included participants from PEI, the number of participants from each province is not known nor are the outlined needs specific to PWID in PEI: the reason being that the data are collapsed across all four Atlantic provinces. It is our intention to complement the existing literature by focussing on PWID in the province of PEI. There does not appear to be any published research that exclusively examines the services needs—harm reduction, medical, detoxification, and methadone treatment services—of PWID within this Canadian province. Importantly, although Jackson et al. and Parker et al. included participants from PEI in their study, the scope of their investigation was limited primarily to harm reduction services, and due to their amalgamation of emergent issues across all four Atlantic provinces, those specific to PWID living in the province of PEI may, ultimately, be obscured [9,11].

### Services for PWID in PEI

**Figure 1:**
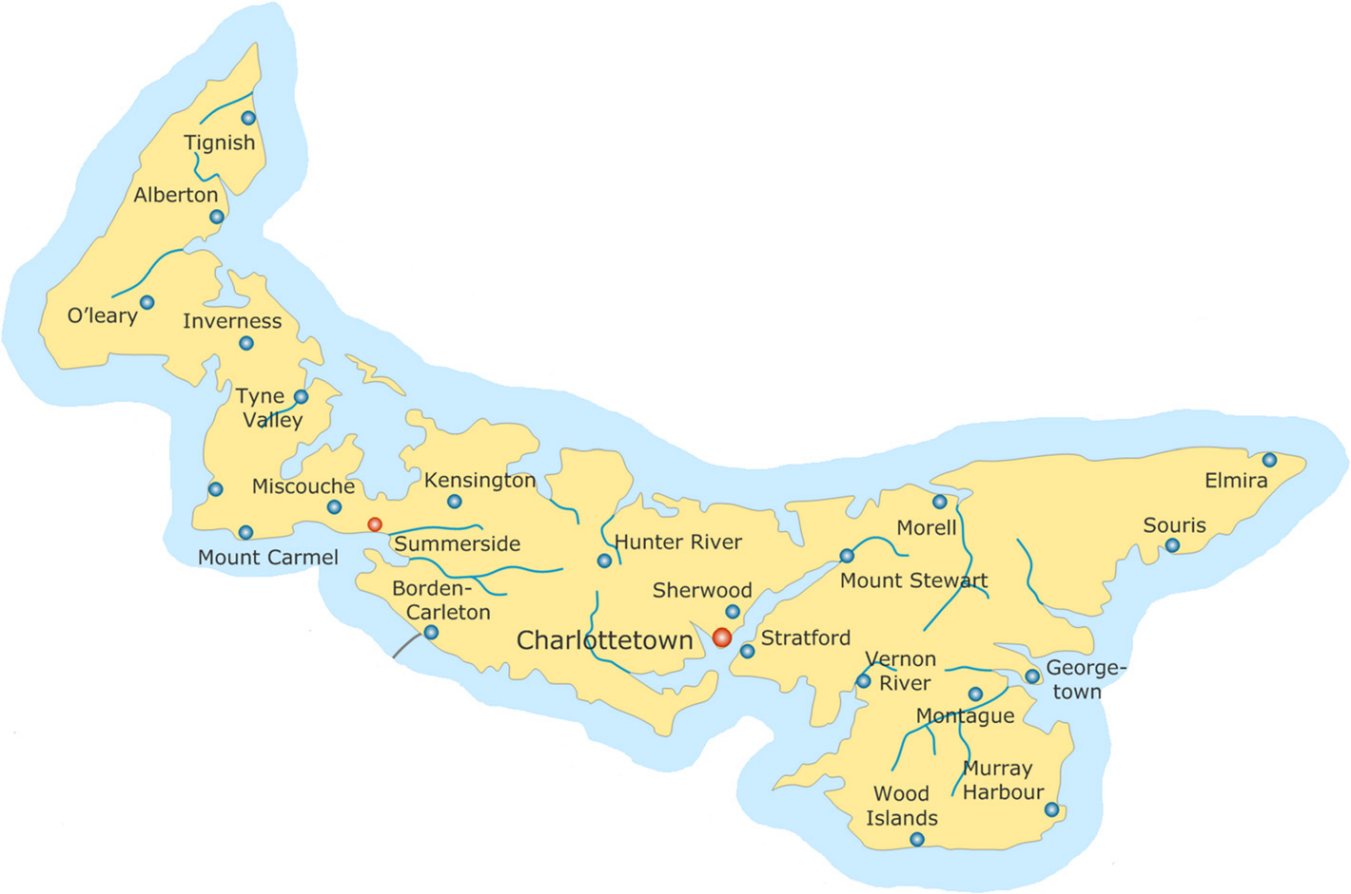
Map of Prince Edward island.

PEI (see Figure 1), located on the east coast of Canada, is the nation's smallest province with a population of approximately 143,800 spread over 5,660 km^2^[12,13]. The context of injection drug use in PEI is difficult to assess given that, when PEI is included in national or regional studies on PWID, the province's data are often not available or are amalgamated with data from the neighbouring province of Nova Scotia [14]. This can be problematic since the other three Atlantic provinces have urban centres with populations far exceeding those in PEI and thus may not be comparable. Apart from the Government of PEI's evaluation of the province's only methadone maintenance treatment programme, the authors found no research relating solely to the experiences of PWID in PEI [15].

In terms of the treatment options for PWID in PEI, the primary location is the Provincial Addictions Treatment Facility located outside the capital city of Charlottetown. The Provincial Addictions Treatment Facility offers a detoxification programme for drug, alcohol, and gambling addictions and contains 25 detoxification beds. The Provincial Addictions Treatment Facility also is the location for the province's methadone maintenance treatment programme. As well, PEI's provincial government offers preventative services through their four syringe exchange centres.

The first syringe exchange centre opened in 2002 in Charlottetown and was operated by the province's AIDS organisation until 2009; after which, the provincial government took over its operation. The provincial government subsequently opened an additional centre in Summerside [16], the second largest city in the province. By 2010 and 2011, respectively, syringe exchange centres were established on the western and eastern ends of the island in an effort to provide access to PWID who were too far from the two existing centralized locations [17,18]. The Charlottetown centre is open Monday to Friday in the afternoons, and the Summerside location dispenses sterile syringes two afternoons per week. The western and eastern locations are each open one afternoon per week. In stark contrast to Vancouver's supervised injection facility that remains open for a period of 18 h per day, the operating hours of the PEI centres appear extremely limited. As PWID often require late-night syringe access, these limitations are problematic [10,19].

## Methods

The present study was conducted in collaboration with AIDS PEI, an organisation that provides support to people living with HIV/AIDS and engages in HIV prevention through harm reduction activities.

### Participants

Participants were recruited using various methods. Firstly, advertisements for the study were sent to organisations that offer services to PWID provincially to post in their offices or to distribute to clients. Further, given their involvement with the study, AIDS PEI staff members assisted in the recruitment of participants by mentioning the study to some of their clients. It should be noted, however, that staff members were not present during the interviews. Advertisements also were placed in downtown Charlottetown and Summerside, the province's two most populous cities. Further, for recruitment purposes and at the invitation of a PWID, the first author attended Narcotics Anonymous meetings throughout the province. At the end of these meetings, individuals were approached to see if they would be interested in participating in the present study. Additionally, all participants and other PWID who were approached were asked if they knew anyone who would fit the eligibility criteria and would be willing to participate. Eligibility criteria for the present study were as follows: (1) self-identification as a PWID, (2) resident of PEI, and (3) 18 years of age or older. Due to the nature of the recruitment methods employed, it is unknown how widely information about the current study was disseminated; therefore, it is difficult to speculate on the number of eligible individuals who heard about the study but chose not to participate.

Following all of the aforementioned recruitment strategies, eight PWID (three females and five males) were interviewed. The female participants ranged in age from 22 to 33 (*M* = 29, SD = 6.35), and male participants ranged in age from 30 to 45 (*M* = 37, SD = 6.95). One participant chose not to specify his age. Six participants were identified as Caucasian and two as Aboriginal-Caucasian. With respect to educational attainment, two participants held a university or college degree, two had completed some college, three had completed high school, and one had completed some high school. One participant was still using at the time of the interview, four participants had injected within the last 12 months, and three participants had gone 2 years or more without using injection drugs. Despite varying lengths of abstinence, all participants self-identified as a PWID and, therefore, were included in the study.

The decision to terminate recruitment after eight participants were interviewed was made only after it was felt that all feasible avenues for accessing participants had been exhausted. After a significant amount of time had elapsed without any new PWID volunteering to participate, despite continued efforts to actively recruit them, a preliminary analysis of the data was conducted to appraise if it would be methodologically sound to conclude interviewing. The data suggested, supported by the first authors' interactions with PWID (participants and non-participants), that saturation had been reached (i.e. the same themes were emerging repeatedly; [20]). Thus, while the authors acknowledged that eight participants may be perceived as a relatively small sample and may limit the ability to generalise to the wider population, Baker and Edwards [21] note that, when conducting interviews with hard-to-access populations, such as PWID, a sample size as small as six can offer extremely valuable insights.

### Procedure

The questions posed during the semi-structured interviews were designed to assess the services that participants currently access or have accessed in the past, the barriers faced when accessing these services, potential means of improving the services that are currently being offered, and whether additional services are needed. PWID agreeing to participate were interviewed at a time and location that was mutually agreed upon. The interviews ranged from 30 to 90 min in duration.

Ethical approval was obtained from the authors' institutional research ethics board. All participants completed written consent forms, which stipulated that their responses would remain confidential and that they had the right to withdraw from the study at any time. As these interviews were conducted in a relatively small region, personally identifying information was removed or changed during data transcription. Consent forms and demographic data were stored separately from the interview transcripts, and to safeguard anonymity, pseudonyms were given to all participants. Pseudonyms were selected using an online random name generator.

### Data analysis

Interviews were audio-recorded and transcribed verbatim. The transcribed data were analysed using thematic coding [22], which is a qualitative technique that facilitates identification of recurrent patterns among interviews [20]. As per Marshall and Rossman's [23] recommendation, the first author read participants' interviews multiple times, thus ensuring an intimate familiarity with the data. Focusing on the types of contextual factors influencing drug-related harm [24,25] and in conjunction with the literature on the needs of PWID [10,14,19], the first author identified preliminary themes based on an initial review of the interview transcripts. As these categories were refined, additional themes emanated from the data inductively. After data reduction, the themes were reviewed and reorganised into overarching categories, thereby facilitating an understanding of the data in its entirety. After coding was completed, the second author reviewed the themes, and any disagreements were resolved through discussion.

### Conceptual framework

The conceptual frameworks of Galea et al. [24] and Rhodes [25,26] were used to guide data analysis in the current study. Both frameworks focus predominantly on how the social context in which PWID seek treatment and harm reduction services influences health behaviours. Galea et al. [24] posit that social policy and regulation affect contextual-, mediating-, and individual-level factors related to drug use behaviour. For instance, if policies were enacted to increase the availability of treatment services for PWID in a specific area, this may, in turn, decrease the number of PWID in that location. The researchers cite a number of key contextual factors associated with drug use behaviour including structural (e.g. service availability), social (e.g. social norms and attitudes), and physical dimensions (e.g. housing). Likewise, Rhodes [26] argues that risk reduction should be approached as a multi-level activity, encompassing not only individual components but also incorporating community and environmental elements. He notes that it is the risk environment, the social and physical spaces that can interact with an individual to increase the likelihood of drug-related harm, which largely defines the success of any policy or intervention. Expanding on the contextual factors put forth by Galea et al., Rhodes includes four types of environment: physical, social, economic, and policy [24,25]. The current study focuses on these contextual factors; specifically, whether existing drug-related services (i.e. syringe exchange centres and treatment facilities) meet the needs of PWID in the province of PEI and whether specific social forces perpetuate the marginalisation of this group.

## Results

### Inadequate or unavailable service provision

All participants reported that current harm reduction strategies and service provision for PWID in PEI were insufficient. Three subthemes were found in relation to the inadequacy or unavailability of service provision: (1) limited access to sterile syringes, (2) lengthy wait times for treatment, and (3) shortage of health care practitioners. Within the subthemes, participants' recommendations for improved services also are identified.

#### Limited access to sterile syringes

A commonly discussed element of the structural environment that shapes drug-related harm concerned participants' access to sterile syringes. Participants had two options available to them when obtaining sterile syringes; they could exchange their used syringes for sterile ones at the syringe exchange centres or they could purchase them at pharmacies. While most participants reported frequenting the province's syringe exchange centres on occasion, all participants indicated that, due to their convenient locations, pharmacies were the outlets most commonly accessed for sterile syringes. However, some participants mentioned their inability to secure sterile syringes when they needed them most. Given the size and population of the cities and towns in PEI, most pharmacies close at 9.00 p.m., with only one Charlottetown location staying open until midnight. Indeed, one participant stated that the limited hours of operation of both syringe exchange centres and pharmacies contributed to the hazardous practice of sharing syringes:

The pharmacy isn't open 24 hours and….the biggest necessity for clean needles is actually during…hours which aren't regular business hours….so there was times that I found myself having to use someone else's syringes and those were times when you know the pharmacies were closed and the needle exchange was closed. (Greg, 30, 2 years without injecting)

All of the participants were aware of the importance of using sterile syringes; however, most of them indicated that this concern did not override their need to use. As a result, all participants reported using their own syringes repeatedly, and a majority reported that, at some point, they had shared syringes with others. Although participants were aware that by reusing their own syringes they would not contract blood-borne infections from others, some participants outlined the deleterious consequences associated with this practice. For example, one participant noted:

I had to use the same [*syringe*s] and I've had them even break off in my arm….[the scars] were great big things. They're finally going down…that was just a mess. I've got marks under my knees, on my feet, my legs, on my boobs, everywhere. (Patricia, 33, 7 months without injecting)

Several participants suggested recommendations for improving access to sterile syringes. One participant recommended that a vending machine selling sterile syringes be considered. He stated:

Have some kind of automatic vending machine and maybe you put in like 25 cents to get one pre-packaged needle or something….to provide access to clean needles at all times. (Greg, 30, 2 years without injecting)

Changes to the policy environment were among the recommendations made by participants. For instance, having PWID act as secondary distributors was believed to facilitate the distribution of sterile syringes and could potentially reduce the stigma encountered when purchasing syringes. Further, distributors could be given additional injection equipment to disseminate to the community. One participant describes how he engaged in secondary distribution and how it provided him the opportunity to educate other PWID:

I distributed [syringes and swabs] in the community, I picked up dirty needles, gave out clean ones, gave out condoms, literature, stuff like that. (Lucas, 45, still using)

Both of the recommendations for improving access to sterile syringes could also accommodate PWID who are unable to access, whether because of geographical distance or timing, one of the province's four syringe exchange centres. In addition, participants also indicated that PWID would benefit from education about safe injection practices. One participant explained that while a ‘clean needle might be used…maybe they'll use a dirty spoon,’ and without adequate information, PWID might not know the myriad ways that infections can be transmitted.

#### Lengthy wait times for treatment

Most participants reported that, once their drug use escalated to a certain point or the consequences of their use became overwhelming, they decided to seek treatment for their drug dependency. Although many of the participants tried to gain access to the services offered by the Provincial Addictions Treatment Facility, a 1- to 2-year waiting period prior to commencing a programme of any kind was typical as evidenced by the following two extracts:

I had actually tried to get on the methadone maintenance programme several times through the…addictions treatment centre….I was unable to get onto… the maintenance programme through them. (Greg, 30, 2 years without injecting)

[The Treatment Facility staff] ask you at some point, “Do you wish to be placed on the methadone list?” You say, “Yes.” They say, “Well, great, we'll probably talk to you in about a year's time.” (Lucas, 45, still using)

These lengthy waiting lists may have dire consequences for the lives of drug-injecting men and women.^b^ In the next excerpt, one participant explains:

Well, if I had have been on the programme, I probably wouldn't have turned to injection. (Daniel, 33, 6 months without injecting)

Below, one participant further attests to these challenges:

[My partner] was trying to get into detox….he tried to get in and they wouldn't let him in. So that, to me, [showed me that there] was something wrong with the programme…He had depression really bad…they all knew he had problems, somebody should have stood up and let him in right away instead of making him wait…If he had have been in detox…he wouldn't be dead [from suicide] right now. (Patricia, 33, 7 months without injecting)

Without timely access to the Provincial Addictions Treatment Facility and it being the only methadone maintenance programme in PEI, participants were compelled to identify other ways of receiving methadone treatment. For instance, some participants were able to get treatment from physicians who could prescribe methadone. One participant explains this process:

My family doctor… made me an appointment for another doctor…that has a license to prescribe methadone to injection drug users as a means of getting them to stop using drugs. There's only four doctors on the Island who have that type of license….That particular doctor said that there was no problem with me getting methadone…he had me on methadone the next day. (Greg, 30, 2 years without injecting)

One participant was forced to leave PEI and go to a more urban city in the neighbouring province of New Brunswick in order to get methadone treatment after having been refused by physicians and having been told that it would be at least 2 years before he could receive treatment from the province's methadone maintenance treatment programme. He explains:

There's a two-year waiting list because there's no funding for anybody, they're not taking patients or anything for it now, cause it's just not in their budget. They're at their limit, they're stretched, there's no resources for it at all….So that's why I chose to [go to New Brunswick]. (Adam, 41, 4 months without injecting)

Adam further states that, after his move to New Brunswick, he ‘got on [methadone] right away’.

With only 25 detoxification beds at the Provincial Addictions Treatment Facility, a limited number of individuals can be admitted at any one time. The lack of readily available services posed a structural barrier for participants. Moreover, the economic environment further perpetuates the inaccessibility of treatment services. Participants who had succeeded in being admitted to the detoxification programme commented that increased funding could be used to establish follow-up services. One respondent described the difficulty abstaining from injection drugs on his own after treatment:

What they do in the detox is dry you out so you're healthy enough…for seven to ten days you're off your medication. After that point, you have to struggle, and go to your meetings, and keep your head down, and keep going ahead, but it's just so hard to do. (Adam, 41, 4 months without injecting)

Implementing a walk-in clinic that would house a detoxification centre as well as counsellors, physicians, and pharmacists also was recommended. By having all of the services in one location, it was perceived as a means of encouraging individuals engaged in treatment to utilise a broader range of services during all steps of their recovery.

#### Shortage of health care practitioners

Several participants discussed how the inadequacy of services and lengthy waiting lists could be related to a shortage of health care practitioners across the province. For instance, Hanna noted, during one of her visits to the Provincial Treatment Facility, that ‘there's a bunch of beds empty.’ This observation is incompatible with participants' accounts of their inability to access expeditious treatment at the facility. Hanna indicated that despite the available infrastructure, there were ‘not enough nurses’ to accommodate full occupancy.

As mentioned, without access to the Provincial Treatment Facility, several participants sought methadone treatment through private physicians. However, given the small number of physicians in PEI who can prescribe methadone, PWID may not be able to access this drug through the Provincial Addictions Treatment Facility or physicians operating independently of the facility:

Call [the doctors in PEI] and see if they're taking any new patients for methadone of which the answer is “no.” (Lucas, 45, still using)

I guess [physicians] have to reject patients because they have too much of a clientele. (Adam, 41, 4 months without injecting)

Participants surmised that increased funding to hire additional staff would lead to reduced wait times at treatment facilities. However, they promptly acknowledged that without greater funding for additional health care practitioners, a feature shaped by the economic environment, these improvements would remain unrealised:

My advice would be to have an open methadone clinic…. but that will never happen cause we're talking about jobs. (Lucas, 45, still using)

### Negativity from health care practitioners

Many participants cited negativity that was directed at them from PEI health care practitioners. Specifically, participants observed two main types of negativity: (1) negativity related to insufficient training of health care practitioners and (2) stigma.

#### Insufficient training of health care practitioners

When participants were asked about their experiences with health care practitioners, they commonly cited the insufficiency of the medical community's training about drug dependency. Firstly, it was felt that part of this insufficiency was related to physicians' and nurses' perceived inability to gauge the appropriate amount of medication to prescribe. Some participants maintained that, when they tried to receive medical service, they were not given enough pain medication due to their status as a PWID, despite their stated need. When seeking treatment for a non-drug-related injury, one participant spoke of how he was forced to endure unnecessary pain because he had admitted to health care workers that he was a PWID:

I was in the hospital…and I told them I was an addict and stuff, like I was clean when I went in, and they had to give me opiates for pain…when I was discharged they told me they could have gave me more for pain but because of me having an addiction, they didn't want to give me more. So they let me suffer. (Daniel, 33, 6 months without injecting)

One respondent, who had received services at the Provincial Addictions Treatment Facility several times after being fast-tracked because of the severity of her drug dependency expressed her frustration in the following way:

[The nurses are] not very good, they don't understand….the nurses right on [the] detox unit, it seems like they don't really know what kind of aches and pains you have in withdrawals. (Hanna, 33, 3 months without injecting)

Recommendations were primarily in regard to the policy environment. That is, numerous participants recommended that the curriculum for health care practitioners be revised to include additional training focussing on working with PWID. They also recommended that guidelines for the provision of drugs be created because it was felt that some physicians prescribe more drugs than are necessary. In hospital situations, it was perceived that health care practitioners allocated drugs in cases when they were unwanted and/or dangerous. One participant recounted a hospital scenario in which her partner was given Dilaudid as part of his treatment, despite both his and her wishes that opiates not be among the drugs provided. She stated:

After we both said that he was a recovering drug user, they should have marked that right on his chart. (Patricia, 33, 7 months without injecting)

Participants feared that these types of omissions might lead non-using individuals to relapse after medical treatment.

#### Perceived stigma from health care practitioners

An element of the social environment that was discussed involved the experience of stigma from health care practitioners. In fact, some participants felt that, because of their status as a known PWID, attempts at seeking any acute medical services were compromised. One participant stated that he had waited in the hospital for over 8 h while trying to obtain medical attention for a drug-related issue. He relayed:

I've heard them say…. “[He] is just a drug addict or a narcotic user, just put them in a corner until later.” (Adam, 41, 4 months without injecting)

Based on participants' accounts, stigma is particularly prevalent when seeking medical treatment for drug-related issues at hospitals. Lucas commentated that he was often treated as a ‘second-class citizen.’ He provided an example from one of his hospital visits:

One nurse called me a frequent flyer one day, the reference being someone who comes to emerg[ency] looking for narcotics. (Lucas, 45, still using)

However, hospitals were not the only location where participants reported being stigmatised because of people's negative attitudes toward PWID. Some participants emphasised the discrimination they experienced when purchasing syringes. Pharmacists and pharmacy employees were perceived to be less accepting of PWID buying syringes in comparison to staff at syringe exchange centres. For instance, one participant recounted:

I had one pharmacist get me before I exited his pharmacy and he said he would appreciate it if I never came into his store again because of what I had just bought at the pharmacy counter. (Lucas, 45, still using)

Also, when PWID are diagnosed with injection-related health problems, the challenges they encounter when seeking medical services may be further intensified. One participant, who is co-infected with HIV and hepatitis C, often experienced isolation from physicians, nurses, and dentists because of his diagnoses. He indicated:

I went to a dentist…and he told the receptionist to close the door and to make sure everything's sterilised in there. [He] just had to take an x-ray of my mouth, didn't even put nothing on me, just against my face and he wanted the whole wall, floor done, the seats, [he] put a biohazard sign on the door….It's just isolation, they treat you like an animal. (Christopher, 7 years without injecting)

## Discussion

Our study identifies key barriers affecting PWID who are looking to safely inject or treat their drug dependency and provides PWID-generated recommendations addressing the barriers, namely sterile syringe access, treatment access, perceived discrimination by health care practitioners and the insufficiency in their training in relation to drug use, and lack of follow-up services. During one-to-one interviews, eight participants responded to questions about the services they typically access or have accessed in the past, the extent to which they can, and wish to, access sterile syringes, their experiences with various treatment services and health care practitioners (e.g. the province's treatment facility and interactions with medical staff, respectively), impediments to receiving treatment, and recommendations for improved sterile syringe access and service provision. The frameworks of Galea et al. [24] and Rhodes [26], with their focus on environmental factors, guided the data interpretation. Challenges obtaining sterile syringes and access to treatment highlight the need for economic and policy changes to the service provision in PEI, while PWID who perceived discrimination, in relation to their receipt of medical care, denotes the presence of social barriers.

When questioned about where they accessed their sterile syringes, most participants reported buying sterile syringes at pharmacies instead of using one of the province's four syringe exchange centres. Due to the syringe exchange centres' limited hours of operation, pharmacies offered significantly greater opportunity to obtain sterile syringes during the later parts of the evening. It should be mentioned, however, that participants cited the hours of *both* the syringe exchange centres and pharmacies as being their biggest challenge when trying to access sterile syringes. Given that syringe exchange centres have limited hours (e.g. only 2.5 h per week for some participants) and pharmacies do not offer 24 h per day services as they do in large cities, a structural barrier such as this may be particularly problematic for PWID in rural or smaller urban centres.

Due in part to the inaccessibility of sterile syringes late at night, all participants reported re-using their own syringes, with six of the eight reporting having shared syringes with other PWID. Although participants were aware of the risks associated with the latter practice, they were less aware of the risks inherent in sharing drug injection paraphernalia such as spoons, water, and filters. This finding is consistent with the research [27] suggesting that, despite having considerable knowledge on safe-injecting behaviour, many PWID continue to engage in risky injection practices. Indeed, four of the participants indicated that, at the time, they did not care about the consequences of either practice (i.e. re-using syringes or sharing them with other PWID).

Participants offered a number of recommendations to improve access to sterile syringes, which included extending the hours of operation at the syringe exchange centres (i.e. ensuring that the centres are operational outside of regular business hours), purchasing vending machines for the distribution of sterile syringes, and organising PWID to serve as secondary distributors. Several of these suggestions for improving syringe access are supported by the literature [28-30]. For example, Islam and Conigrave found that syringe-dispensing machines are effective means for distributing syringes to those who are unable, or unwilling, to frequent syringe exchange centres or pharmacies [31], and they can be incorporated into the existing distribution tactics of syringe exchange programmes. Although population density is relatively low in PEI, syringe-dispensing machines could be erected in the downtown regions of the most urban areas of the province, Charlottetown and Summerside, where it is likely that PWID are concentrated. Researchers also found that secondary distribution by other PWID or drug dealers could increase the use of sterile syringes among PWID who do not procure sterile equipment through formal distribution services [19,32]. Syringe exchange programmes also can facilitate secondary distribution by furnishing providers with biohazard containers for used syringes and not limiting the number of syringes provided. According to Klein, PWID may prefer dispensing machines or secondary distribution as means of maintaining their anonymity [33]. Further, these methods may be of particular importance for PWID in smaller communities who cannot purchase sterile syringes from pharmacies without suspicion from community members [31,33].

In a federal study examining drug dependency programmes and policies, the Canadian Mental Health Association reported that ‘about half of the adult population who need services [including treatment designed to treat or manage drug dependence] must wait for eight weeks or more’ ( [34], p. 161). Based on this estimate, it appears that PWID in PEI are facing comparatively longer wait times prior to starting a provincially funded treatment programme (i.e. periods of time in excess of 2 years) than PWID situated in other provinces. Indeed, seven of the eight participants who had received treatment from the Provincial Addictions Treatment Facility at some point in the past, indicated that the long waitlists was the biggest challenge to starting treatment, and a majority reported that their drug use typically intensified when treatment was inaccessible. Although some participants were able to access private clinics that had physicians who would prescribe methadone, most of our participants indicated that there are too few physicians in PEI (i.e. four in total) who can write methadone prescriptions, and at the time of our interviews, these physicians were not taking new patients. Based on these barriers, participants recommended that the provincial government increase funding for treatment services for PWID, a recommendation that may not be easily implemented. For instance, Gustafson et al. caution that small regions may not have the economic resources to increase funding, and therefore, initiatives such as these may not be feasible [10].

The nature of the medical care received by participants also was cited as a problem. Participants reported that doctors and nurses did not know enough about drug dependency and either dispensed narcotic drugs too liberally or did not supply enough narcotics for pain relief when a patient was identified as a PWID. Several participants in the present study reported that they were the recipients of verbal (e.g. name-calling) and non-verbal discriminatory behaviour (e.g. receiving no service) from health care practitioners and pharmacists. In their examination of incidents of discrimination encountered by PWID in Australia, Day et al. found that 37% of these events occurred in health care settings and more than half resulted in refusals of service [35].

### Limitations and future directions

The present study advances understanding of the current atmosphere for PWID in PEI and offers PWID-generated recommendations as to what can be done to improve services for this population. Our interviews suggest that the current services for PWID do not meet the demand for treatment; this is valuable information given that no studies of this kind have been conducted in the province of PEI to date. However, since only eight PWID were interviewed, further research is warranted. Despite assurances of confidentiality and anonymity, most PWID were unwilling to participate in the present study because of their apprehension when asked to discuss their injection behaviour with someone associated with a service provision organisation due to fear of negative reprisals (e.g. loss of social assistance or child custody). Researchers should ensure that every effort is made to provide reassurance to PWID that their participation will not result in these deleterious consequences. One method to encourage increased participation among PWID may be the formation of an interdisciplinary team. For instance, the addition of a social worker may have assuaged the fears of those PWID who were apprehensive about repercussions related to their social assistance status or their parental rights, or the inclusion of a lawyer could have offered legal protection to those who were concerned about reporting on their illegal behaviour. Also, research teams that are unaffiliated with organisations providing services to PWID could potentially increase willingness to participate due to a greater level of perceived anonymity. Service providers in Atlantic Canada indicate that the preservation of PWID's anonymity may be particularly important for those in less populated regions, making the need to provide additional assurances of confidentiality and security critical when recruiting this population [36].

The number of PWID who agreed to participate also may have been low given that only Canadian 5$ was provided to those who were interviewed. By providing a greater monetary incentive, it is possible that more individuals would be willing to participate. Further, incentives such as free syringes, assistance with travel to and from the interview, and other gift-related alternatives (grocery cards, gift cards, products, and so forth) could be offered. Incorporating appropriate incentives into the research protocol could promote greater interest in participating. Additionally, the sampling procedures may have affected the themes that were generated. Since participants were recruited primarily through harm reduction and treatment organisations, this may be why only one participant reported currently injecting drugs (and all other participants reported some length of sobriety). Future research concentrating on PWID who are not utilising treatment or harm reduction services could provide additional information about the needs of PWID in PEI.

It also should be acknowledged that the primary researcher, who conducted the interviews, is not a current or former PWID and may not have been as readily accepted into the community or as trusted as an insider may have been. In an effort to recruit and build rapport with PWID, researchers might consider a team approach wherein a current or former PWID actively participates during interview sessions. Not only would this strategy likely improve participants' general comfort, but it also may assist the elucidation of themes that are not as easily arrived at when PWID are engaged in discussion with an outsider. However, an interviewer who may be less familiar with the *minutia* of injection drug use is more likely to probe for details that are presumed known by the insider. Some researchers suggest that a research team including both an insider and outsider is ideal [37,38].

Participants also may have been selective with the information that they provided in order to correspond to the perceived expectations of the researcher. Male participants, in particular, may have tailored their answers in response to being interviewed by a woman. Therefore, if researchers choose to use the team approach in future studies, it may be useful to have interviewers of different genders or to match the gender of the participant with the interviewer. This approach also may highlight any gender differences that exist between the needs and lifestyles of male and female PWID. For instance, research has found that female PWID tend to participate in more syringe sharing, likely due to their larger social networks; however, they also engage in more protective behaviours such as carrying clean syringes and frequenting syringe exchange centres [39,40]. While not a focus of the present study, our interviews revealed differences between men and women insofar as their initiation to injection drugs, the methods they use to obtain drugs, how they secure treatment services, and their fears related to speaking to service providers. Gender-related differences such as these should be explored in small urban centres in greater detail.

Finally, ongoing research is needed on PWID in PEI due to the continuous changes in service provision. The present study was conducted in 2010, prior to the establishment of the syringe exchange centre on the western side of the province. Additional research could determine how this new centre may affect sterile syringe access in PEI and how trends in injection drug use and PWID's service needs change over time.

## Conclusions

While PWID in the present study report encountering barriers similar to those described by PWID in large urban centres, the challenges faced by PWID in this area appear to be intensified due to the limitations of a smaller region's harm reduction, treatment, and health care services. The present study revealed that the current services for PWID are not adequately meeting their demand for treatment. Researchers should continue investigating novel channels through which interventions could be provided to PWID who, due to the province of PEI's funding constraints, cannot benefit from the services offered in larger cities. Finally, when creating new initiatives or improving current services for PWID in smaller urban centres, policy makers should be cognisant that their experiences may differ from PWID in more populated urban centres.

### Endnotes

^a^St. John's, Newfoundland and Labrador is located on the island of Newfoundland and has a population of 196,966 (spread over 805 km^2^) [41]. Newfoundland and Labrador, Canada's easternmost province, has the country's highest unemployment rate and is one of its most economically depressed provinces [42]. Recent environmental and economic restructuring has resulted in increased job loss and emigration from the province [43].

^b^While wait time data for PEI's drug dependency services are not available, the Government of Nova Scotia, in a neighbouring province to PEI, reports that nine out of ten patients wait between 26 to 54 days (depending on their health district) to receive structured residential treatment [44].

## Competing interests

The authors declare that they have no competing interests.

## Authors’ contributions

JMM designed the study, managed the data collection, coded and analysed the data, and wrote large portions of the manuscript. MAM assisted with the manuscript development, data analysis and interpretation, and wrote portions of the manuscript. Both authors read and approved the final manuscript.
